# Validation of myocardial perfusion and coronary flow reserve in rats using spin-labeling gradient echo imaging with a fluorescent microsphere technique as standard of reference

**DOI:** 10.1186/1532-429X-11-S1-P148

**Published:** 2009-01-28

**Authors:** Alexis Jacquier, Franck Kober, Soksithikun Bun, Patrick J Cozzone, Monique Bernard

**Affiliations:** CRMBM, UMR CRNS 6612, Marseille, France

**Keywords:** Myocardial Perfusion, Isoflurane, Myocardial Blood Flow, Coronary Flow Reserve, Arterial Spin Label

## Introduction

Cardiac arterial spin labeling (ASL) MRI might become an important technique for quantitative mapping of myocardial blood flow in humans. A previously proposed gradient echo ASL technique has provided high spatial resolution but without external validation against microspheres technique.

## Purpose

The purpose of this study was to validate *in vivo* myocardial perfusion measurements and coronary flow reserve by gradient echo arterial spin labeling (ASL) MRI in rats anesthetized with isoflurane against fluorescent microspheres technique as standard of reference.

## Methods

Male Wistar rats (weight = 200–240 g, n = 21) were anesthetized with 2.1% isoflurane added to 1 l/min of pure O_2_. Heart rate, breath rate, temperature, blood oxygen saturation and arterial blood pressure were recorded. In 7 rats, myocardial perfusion was assessed on a Bruker Biospec 4.7 T horizontal MRI system using an ECG- and respiration-gated IR gradient-echo technique (resolution = 234 × 468 μm^2^, TE = 1.52 ms, slice thickness 3 mm, acquisition time 25 min at 350 bpm) at rest and during adenosine infusion (140 μg/kg/min). In the 14 other animals, under the same physiologic conditions, a mixture containing 200,000 fluorescent microspheres (Yellow, 15 ± 0.1 μm; Triton, San Diego, CA, USA) was injected into the left ventricle at rest in 7 animals and during adenosine infusion in 7 other animals. The animals were killed by instantaneaous injection of pentobarbital. Hearts were harvested and samples were processed for fluorescence spectroscopy. A two-tailed unpaired Student's t-test was used to compare groups, All values are given as mean ± SD.

## Results

**Figure 1:**
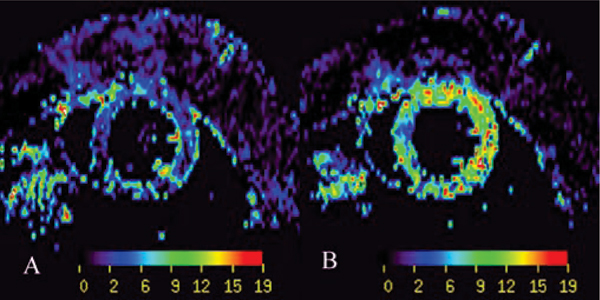
Figure 1

There was no statistical difference between myocardial perfusion at rest assessed with ASL (6.5 ± 1.4 ml/g/min; Figure 1a) and with fluorescent microspheres (5.9 ± 2.3 ml/g/min; P = 0.5). During adenosine infusion there was no statistical difference between myocardial perfusion measured using ASL technique (11.9 ± 1.6 ml/g/min; Figure 1b) and using fluorescent microsphere technique (13.1 ± 2.1 ml/g/min; P = 0.6). Coronary flow reserve was calculated as 2.3 ± 1.1 using ASL. There was no significant difference between groups in terms of heart rate (400 ± 20 bpm), breath rate (50 ± 12/min), temperature (36.9 ± 0.1°C), O2 saturation (98 ± 1%) or mean blood pressure (9.8 ± 0.3 mmHg) at rest. Under adenosine infusion a tendency to heart rate increase was measured (421 ± 21 bpm; P = ns). Group standard deviation was lower with MRI than with microspheres. These values obtained under isoflurane anesthesia are higher than previously reported values under pentobarbital. They confirm capillary vasodilation by isoflurane.

## Conclusion

Non-invasive gradient echo ASL MRI provides accurate and reliable myocardial perfusion maps and assessment of coronary flow reserve with high spatial resolution. This study has provided validation of gradient echo ASL against an external gold standard technique.

